# Total Synthesis of Fellutamide B and Deoxy-Fellutamides B, C, and D

**DOI:** 10.3390/md11072382

**Published:** 2013-07-08

**Authors:** Andrew M. Giltrap, Katie M. Cergol, Angel Pang, Warwick J. Britton, Richard J. Payne

**Affiliations:** 1 School of Chemistry, The University of Sydney, NSW 2006, Australia; E-Mails: andrew.giltrap@sydney.edu.au (A.M.G.); katie.cergol@sydney.edu.au (K.M.C.); 2 Centenary Institute of Cancer Medicine and Cell Biology, Newtown, NSW 2042, Australia; E-Mails: a.pang@centenary.org.au (A.P.); warwick.britton@sydney.edu.au (W.J.B.); 3 Sydney Medical School, The University of Sydney, NSW 2006, Australia

**Keywords:** marine peptides, peptide synthesis, lipopeptide, *Mycobacterium tuberculosis*, proteasome inhibitors

## Abstract

The total syntheses of the marine-derived lipopeptide natural product fellutamide B and deoxy-fellutamides B, C, and D are reported. These compounds were accessed through a novel solid-phase synthetic strategy using Weinreb amide-derived resin. As part of the synthesis, a new enantioselective route to (3*R*)-hydroxy lauric acid was developed utilizing a Brown allylation reaction followed by an oxidative cleavage-oxidation sequence as the key steps. The activity of these natural products, and natural product analogues was also assessed against *Mycobacterium tuberculosis* *in vitro*.

## 1. Introduction

**Figure 1:**
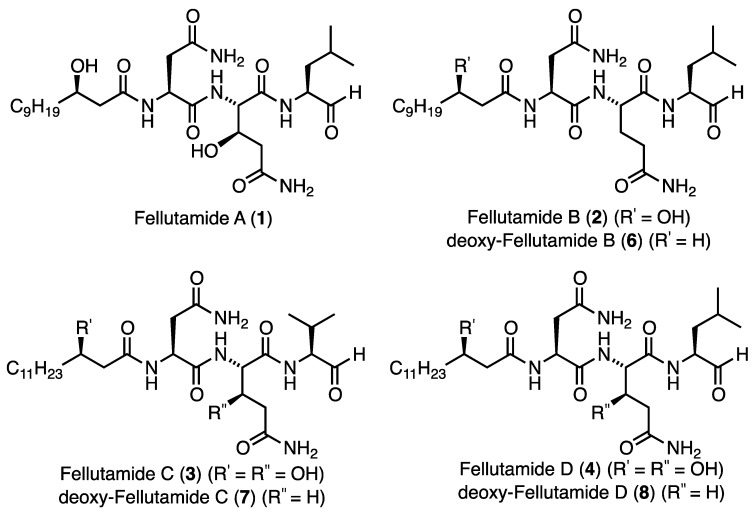
Fellutamides A–D (**1**–**4**), and deoxy-fellutamides B (**6**), C (**7**), and D (**8**).

Fellutamides A–D (Figure 1, **1**–**4**) are a family of marine-derived lipopeptide natural products, characterized by a *C*-terminal aldehyde and a (3*R*)-β-hydroxy alkanoate tail (Figure 1). Fellutamides A (**1**), C (**3**) and D (**4**) contain the non-ribosomal amino acid β-l-*threo*-hydroxy-glutamine, while fellutamides A (**1**) and B (**2**) both possess a β-hydroxylated fatty chain amide derived from (3*R*)-hydroxy lauric acid (**5**).

Fellutamides A (**1**) and B (**2**) were first isolated by Shigemori and co-workers in 1991 from the fungus *Penicillium fellutanum*, found in the gastrointestine of the Candystripe Cardinalfish *Apogon endekataenia*, located off the coast of Japan [1]. Both of these compounds were found to be cytotoxic against human epidermoid carcinoma KB, exhibiting half maximal inhibitory concentration (IC_50_) values of 0.5 μg/mL and 0.7 μg/mL for fellutamide A (**1**) and fellutamide B (**2**), respectively. From this family of natural products only the total synthesis of fellutamide B (**2**) has been achieved which was reported by Crews and co-workers in 2006 [2]. In 2011, Xu and co-workers reported the isolation of fellutamides C (**3**) and D (**4**) from an undescribed species of *Metulocladosporiella*, a fungus isolated from Equatorial Guinean soil [3]. It was reported that the cellular target of fellutamides C (**3**) and D (**4**) is the fungal proteasome, and these natural products were found to be active against a number of *Candida* fungal species with minimal inhibitory concentration (MIC) values between 2 and 32 μg/mL. Interestingly, fellutamide C (**3**) was consistently more potent than fellutamide D (**4**). In addition, screening against Bortezomib sensitive (PC-3) and insensitive (A549) human cancer cell lines revealed that these compounds were potent inhibitors of the PC-3 cell line with no inhibition of the A549 cell line.

Since their isolation, fellutamides A (**1**) and B (**2**) have been found to have a number of other interesting activities, across a range of biological systems [4]. In particular, fellutamide B (**2**) is a potent inhibitor of the *Mycobacterium tuberculosis* proteasome with an inhibition constant (*K*_I_) of 6.8 nM against the *M. tuberculosis* 20S core particle (CP). In fact, fellutamide B (**2**) is the most potent peptide inhibitor of the *M. tuberculosis* proteasome reported to date [5]. Additionally, fellutamide B (**2**) is also a potent inhibitor of the human 20S CP with a *K*_I_ of 11.5 nM [5]. It has since been discovered that fellutamide B (**2**) binds to the *M. tuberculosis* 20S proteasome via a different mechanism to the human 20S proteasome [5]. As such, it can be envisaged that modifications to the structure may result in higher affinity and selectivity for the *M. tuberculosis* proteasome. Two potent irreversible oxathiazol-2-one inhibitors of the *M. tuberculosis* proteasome were recently elucidated from a high throughput screen of 20,000 compounds [6]. These compounds have been shown to cross the cell wall and inhibit non-replicating *M. tuberculosis in vitro*, which may therefore provide inspiration for the development of new TB drug leads which operate via novel modes of action.

**Figure 2:**
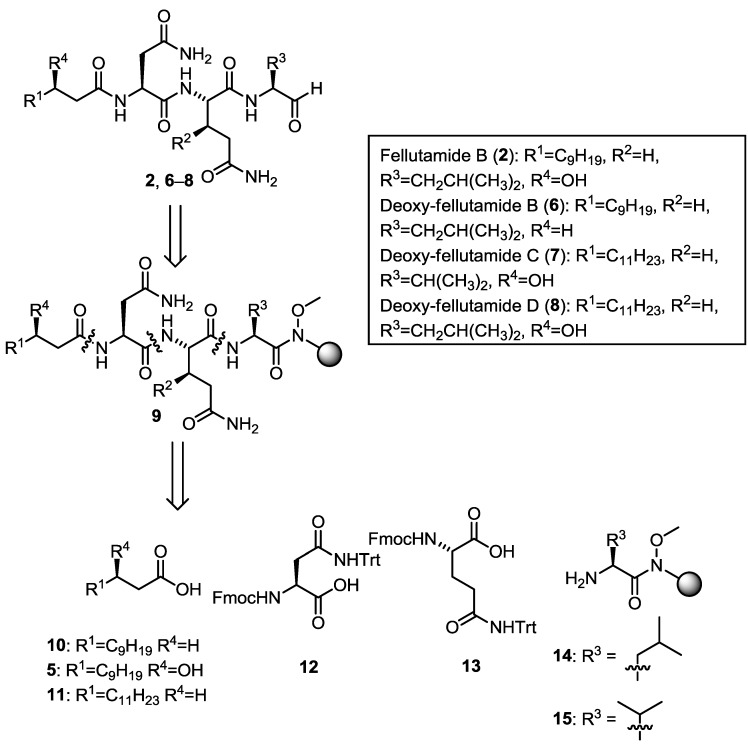
Retrosynthetic analysis for Fellutamide B (**2**) and deoxy-analogues **6**–**8**.

Given the interesting structural features and biological activities of the fellutamide natural products, particularly as inhibitors of the *M. tuberculosis* proteasome, we sought to develop a general synthesis of fellutamide B (**2**) and the deoxy-fellutamide analogues **6**–**8** (Figure 2). Additionally, we were interested in the potential of these compounds as inhibitors of *M. tuberculosis* growth and in the future, as *M. tuberculosis* proteasome inhibitors. Access to these compounds may help to establish the necessity of the hydroxyl groups for binding to the proteasome and for activity against *M. tuberculosis* and lead to the development of selective *M. tuberculosis* proteasome inhibitors as potential anti-tubercular agents.

## 2. Results and Discussion

### 2.1. Synthetic Plan

We envisaged that fellutamide B (**2**) and deoxy-fellutamide analogues **6**–**8** could be synthesized by solid-phase peptide synthesis on Weinreb amide resin [7,8,9] which upon reduction would afford either the *C*-terminal leucinal, present in fellutamide B (**2**), and analogues (**6** and **8**) or a valinal moiety, found in deoxy-fellutamide C (**7**) (Figure 1). The *C*-terminal aldehyde is one of the most interesting structural features present in the fellutamide natural product family, as it is essential for the inhibitory activity of fellutamide B (**2**) against the *M. tuberculosis* proteasome [5]. Due to the inherent reactivity of this functional group, we envisioned that the aldehyde would ideally be introduced in the final step of the synthesis, without the need for subsequent purification. As such, we planned to introduce the aldehyde functionality from resin bound Weinreb amide precursors (general structure **9**) which could be assembled following standard solid-phase peptide synthesis (SPPS) protocols, starting with the fragments **5**, **10**–**15**, which would provide access to fellutamide B (**2**) as well as deoxy-analogues **6**–**8** (Figure 2). The (3*R*)-hydroxy lauric acid (**5**) fragment (R^1^ = C_9_H_19_ and R^4^ = OH) required for the synthesis of fellutamide B (**2**) is not commercially available and, as such, required preparation. 

#### Synthesis of (3*R*)-Hydroxy Lauric Acid (**5**)

**Scheme 1:**
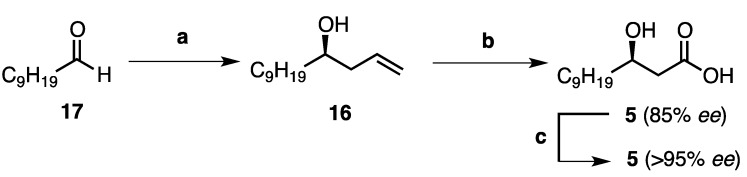
Synthesis of (3*R*)-hydroxy lauric acid (**5**). Reagents and conditions: (**a**) (i) (−)-B(Ipc)_2_(allyl) [(−)-**18**], Et_2_O, −100 °C; (ii) NaOH, H_2_O_2_, reflux to r.t., 83%, 85% *ee*; (**b**) (i) OsO_4_, NMO, (CH_3_)_2_CO, H_2_O, r.t.; (ii) NaIO_4_, THF, H_2_O, r.t.; (iii) NaClO_2_, NaH_2_PO_4_, *t*-BuOH, H_2_O, r.t. (77%, 85% *ee*, over three steps); (**c**) (i) HN(C_6_H_11_)_2_, MeCN, r.t. (recrystallised 3 times from MeCN); (ii) 1M HCl, 75%, >95% *ee*.

Although there have been a number of syntheses of (3*R*)-hydroxy alkanoic acids reported in the literature to date, [10,11] we proposed a shorter route whereby the desired acid **5** could be accessed from the chiral homoallylic alcohol **16** via an oxidative cleavage-oxidation sequence (Scheme 1). Homoallylic alcohol **16** in turn could be prepared via an enantioselective allylation reaction as the key stereochemistry determining step from the commercially available aldehyde, decanal (**17**). To this end, decanal (**17**) was treated with the chiral boron reagent (−)-*B*-allyl-diisopinocampheylborane [(−)-B(Ipc)_2_(allyl)] [(−)-**18**] which was preformed from (–)-*B*-methoxy-diisopinocampheylborane and allyl magnesium bromide at −78 °C (See Supplementary Information). After removal of the resulting magnesium salts by filtration under an inert atmosphere, decanal (**17**) was added to the solution of reagent (−)-**18** at −100 °C. Oxidation of this adduct by addition of basic hydrogen peroxide liberated the free homoallylic alcohol **16**, which was isolated in excellent yield (83%, 85% *ee*, Scheme 1) [12]. The absolute configuration was confirmed by comparison with the literature value for this compound (+2.9, *lit.* +11.2) [13], and the enantiomeric excess was determined from the corresponding Mosher’s ester of **16** (See Supplementary Information) [14]. Furthermore, dihydroxylation of the olefin **16** employing catalytic osmium(VIII) tetroxide and *N*-methylmorpholine-*N*-oxide, and subsequent oxidative cleavage using sodium periodate provided the intermediate aldehyde, which was subjected to Pinnick oxidation conditions to afford the desired acid **5** in good yield (77%, Scheme 1). Finally, recrystallization of the corresponding dicyclohexylammonium salt of acid **5** from acetonitrile (3 times) following the procedure of Tai and co-workers [15] led to enantiomeric enrichment, providing the acid **5** in 58% overall yield, and >95% *ee*, as determined from the Mosher’s ester of the corresponding methyl ester of **5** (See Supplementary Information).

**Scheme 2:**
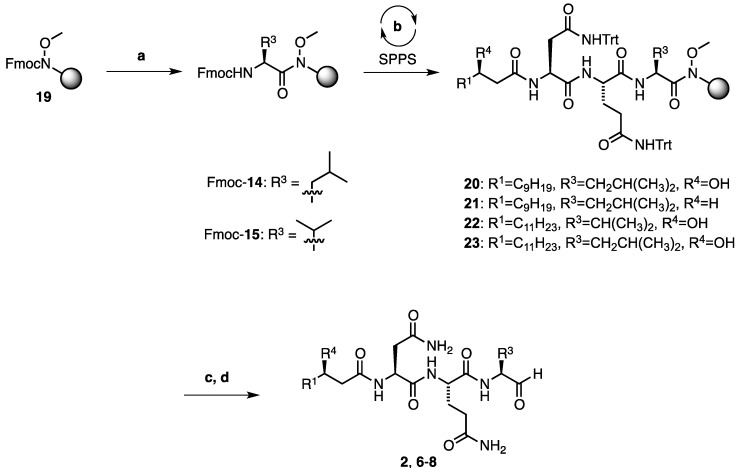
Fmoc-SPPS of resin bound intermediates **20**–**23**. Reagents and conditions: (**a**) Deprotection: 10 vol% piperidine/DMF, 2 × 3 min Coupling: 5 eq Fmoc-Leu-OH or Fmoc-Val-OH, 5 equiv. HATU, 10 equiv. *i*Pr_2_EtN, 2 × 6 h; (**b**) (One cycle) Deprotection: 10 vol% piperidine/DMF, 2 × 3 min; Coupling 1 [Fmoc-Gln(Trt)-OH and Fmoc-Asn(Trt)-OH]: 4 eq AA, 4 eq PyBOP, 8 eq NMM, 1–2 h; or Coupling 2 (for fatty acid **5**, **10**, or **11**): 1.2 eq acid, 1.2 eq HATU, 2.4 eq *i*Pr_2_EtN; and Capping: 10 vol% acetic anhydride/pyridine, 2 min; (**c**) TFA:TIS:H_2_O, (9:0.5:0.5, v/v/v), 1 h; (**d**) LiAlH_4_, THF, 0 °C, then KHSO_4_, NaKTartrate, 0 °C to r.t.

### 2.2. Total Synthesis of Fellutamide B *(**2**)* and Deoxy-Fellutamides B *(**6**)*, C *(**7**)*, and D *(**8**)*

With enantiopure (3*R*)-hydroxy lauric acid (**5**) in hand (Section 2.1, Scheme 1), the synthesis of fellutamide B (**2**) and the deoxy-fellutamide analogues **6**–**8** was next undertaken (Scheme 2). Beginning with Weinreb amide resin **19** and after Fmoc-deprotection, either Fmoc-protected leucine or Fmoc-protected valine was coupled onto the resin using 2-(1*H*-7-azabenzotriazol-1-yl)-1,1,3,3-tetramethyluronium hexafluorophosphate (HATU) as the coupling reagent and *N*,*N*-diisopropylethylamine as the base. The couplings were carried out twice for extended times (6 h) to facilitate complete coupling to the secondary Weinreb amine. After deprotection of the resulting *N*-terminal Fmoc group of Fmoc-protected **14** or **15**, resin-bound precursors **20**–**23** were synthesized following standard SPPS protocols (Scheme 2), whereby fatty acids **5**, **10**, or **11** were coupled in the final step. After deprotection of the trityl protecting groups of **20**–**23** under acidolytic conditions, the resulting resin-bound peptides were dried and swelled in anhydrous tetrahydrofuran before treatment with LiAlH_4_ solution to liberate the free aldehyde functionalities and afford the desired peptide products **2**, **6**–**8** which were produced in good yield (44%–60%) over the six steps (Table 1).

**Table 1:** Yields of fellutamide B (**2**) and deoxy-fellutamides B, C, and D (**6**–**8**) after cleavage from resin based on the original loading of the Weinreb amide resin (6 steps).

Compound	R^1^	R^3^	R^4^	Yield (%)
Fellutamide B (2)	C_9_H_19_	CH_2_CH(CH_3_)_2_	OH	55
Deoxyfellutamide B (6)	C_9_H_19_	CH_2_CH(CH_3_)_2_	H	44 (24% after purification)
Deoxyfellutamide C (7)	C_11_H_23_	CH(CH_3_)_2_	OH	53
Deoxyfellutamide D (8)	C_11_H_23_	CH_2_CH(CH_3_)_2_	OH	60

Gratifyingly, the ^1^H NMR data obtained for synthetic fellutamide B (**2**) matched that of the isolated material with the exception of one signal in the ^13^C NMR spectrum (See Supplementary Information) [1]. Furthermore, fellutamide B (**2**) and deoxy-fellutamide C (**7**) and D (**8**) were of sufficient purity for biological testing without the need for purification (up to 95% purity). However, deoxyfellutamide B (**6**) was purified by reverse-phase HPLC which provided the desired product with no observable epimerisation of the *C*-terminal aldehyde, despite the presence of TFA in the eluent. 

### 2.3. Testing of Fellutamide B *(**2**)* and Deoxy-Fellutamides B *(**6**)*, C *(**7**)*, and D *(**8**)* against *Mycobacterium tuberculosis*

Fellutamide B (**2**) and the three deoxy-fellutamides **6**–**8** were tested against replicating virulent *M. tuberculosis* (H37Rv) *in vitro*. Unfortunately these compounds did not demonstrate any inhibitory activity at concentrations up to 1 mM. Given that the proteasome is essential for resistance to reactive nitrogen species, which are induced following T cell activation of infected macrophages [16], we next screened **2** and **6**–**8** in an *in vitro* model of dormant (non-replicating) *M. tuberculosis* [17]. This was achieved by treating *M. tuberculosis* cultures with the diethylenetriamine-nitric oxide (DETA-NO) adduct as a nitric oxide source during incubation with **2**, **6**, **7** or **8**. Unfortunately these compounds also failed to inhibit the growth of the bacteria at concentrations up to 1 mM in the presence of sub-lethal concentrations of NO. Given that the oxathiazol-2-one class of potent *M. tuberculosis* inhibitors were able to inhibit the growth of *M. tuberculosis* under these conditions *in vitro* [6], this result may suggest that fellutamide B (**2**) and the deoxyfellutamides **6**–**8** are not capable of crossing the cell wall of *M. tuberculosis* and, as such, do not exhibit any measurable *in vitro* activity. Future work in our laboratories will involve assessing the activity of these synthetic fellutamides against the *M. tuberculosis* proteasome, the results of which will be reported in due course. 

## 3. Experimental Section

### 3.1. General Experimental Methods

All reactions were conducted under an atmosphere of argon or nitrogen unless otherwise stated, or if water was present in the reaction. Commercially available chemicals were used as purchased or purified by standard methods where necessary according to Armarego and Chai [18]. The following temperature baths were used: ice/water/salt (0 °C), MeCN/dry ice (−42 °C), acetone/dry ice (−78 °C), diethyl ether/dry ice (−100 °C). Silicon oil baths were used for high temperature reactions. THF was distilled over sodium wire and benzophenone. CH_2_Cl_2_ was distilled over calcium hydride. Other anhydrous solvents were purchased from Sigma-Aldrich. 

NMR spectra were recorded at 300 K, unless otherwise stated, using Bruker Avance DRX200, DRX300, DPX400 or DPX500 spectrometers at a frequency of 200.1, 300.2, 400.2 or 500.2 MHz respectively. ^1^H-NMR chemical shifts are reported in parts per million (ppm) and are referenced to solvent residual signals: CDCl_3_ (δ = 7.26 ppm), C_6_D_6_ (δ = 7.16 ppm) and *d*_6_-DMSO (δ = 2.50 ppm). ^1^H-NMR data is reported as chemical shift (δ_H_), multiplicity (s = singlet, d = doublet, t = triplet, q = quartet), coupling constant (*J* in Hz), relative integral and assignment where possible with the help of COSY and HMBC experiments where necessary. ^13^C-NMR chemical shifts are reported in parts per million and were referenced to the solvent residual signals: CDCl_3_ (δ = 77.16 ppm) and *d*_6_-DMSO (δ = 39.52 ppm).

Low resolution mass spectra (LRMS) were recorded on a Finnigan LCQ Deca ion trap mass spectrometer. High resolution mass spectra (HRMS) were recorded on a Bruker 7T Fourier Transform Ion Cyclotron Resonance (FTICR) mass spectrometer. Melting points were recorded using a Stanford Research Systems Optimelt Automated Melting Point System. Infrared (IR) absorption spectra were recorded on a Bruker Alpha Spectrometer with attenuated total reflection (ATR) capability and were processed with Opus 6.5 software. Optical rotations were measured at 23 °C at 589 nm (Na D line) using a Perkin Elmer Model 341 polarimeter or an Optical Activity PoLAAR 2001 polarimeter, concentrations (*c*) in g/100 mL. 

Thin layer chromatography was carried out on Merck Kieselgel 60 F_254_ pre-coated aluminium sheets. Compounds were visualized by UV light at 254 nm or by staining with vanillin, or phosphomolybdic acid. Flash chromatography was carried out using 230–400 mesh Kieselgel 60 silica get with solvents as described. Ratios of solvent systems used for flash chromatography are expressed in v/v as specified. Preparative reverse phase HPLC was performed using a Waters 2535 Multisolvent Delivery System with a Waters 2489 UV/visible detector operating at 245 and 254 nm. A Waters SunFire™ series C18 column (150 × 19 mm, 5 μm particle size) was used with a flow rate of 7 mL min^−1^. Compounds were loaded in DMSO and eluted with 0.1% TFA in water (solvent A) and 0.1% TFA in MeCN (solvent B) using a linear gradient of 0%–100% B over 40 min. LC-MS was performed on a Shimadzu LCMS 2020 with LC-20AD Pump and a SPD-20A detector on a Waters SunFire™ series C18 column (150 × 2.1 mm, 5 μm particle size), at a flow rate of 0.2 mL min^−1^. All separations involved a mobile phase of 0.1% formic acid in water (solvent A) and 0.1% formic acid in MeCN (solvent B) using a linear gradient of 0%–100% B over 10 or 30 min.

### 3.2. (3*R*)-Hydroxy Lauric Acid *(**5**)*

#### 3.2.1. (4*R*)-Tridec-1-en-4-ol (**16**)

To a solution of (−)-B(Ipc)_2_OMe (6.01 g, 19.2 mmol) in Et_2_O (70 mL) at −78 °C was added allyl magnesium bromide (19.2 mL, 19.2 mmol, 1 M in Et_2_O) dropwise over 20 min and left to stir at this temperature for 10 min. The reaction mixture was then warmed to room temperature over 2 h, and then cooled to −100 °C. Distilled decanal (**17**) (2.41 mL, 12.8 mmol) in Et_2_O (13 mL) was added dropwise by cannula and the reaction mixture was left to stir at −100 °C for 1.25 h and then warmed to room temperature over 1.25 h. The mixture was quenched at 0 °C with 30% v/v aqueous H_2_O_2_ (15 mL) and aqueous 3 M NaOH (20 mL) and heated to reflux for 1 h, and then stirred at room temperature for 16 h. The mixture was poured onto water (100 mL) and extracted with Et_2_O (3 × 100 mL). The combined organic layers were washed with saturated aqueous NaHCO_3_ (100 mL), dried (Na_2_SO_4_), filtered and concentrated *in vacuo*. The crude product was purified twice by flash chromatography (eluent: 5:95 v/v EtOAc/hexanes) and (eluent: 3:97 v/v→5:95 v/v EtOAc/hexanes) to yield homoallylic alcohol **16** as a colourless oil (2.10 g, 83%). 

^1^H-NMR (CDCl_3_, 400 MHz) δ (ppm) 5.89–5.77 (m, 1H, OCCH_2_C*H*CH_2_), 5.17–5.13 (m, 1H, OCCH_2_CHCH*H*), 5.13–5.10 (m, 1H, CH_2_CHC*H*H), 3.68–3.60 (m, 1H, C*H*OH), 2.34–2.26 (m, 1H, OCHCH*H*), 2.19–2.09 (m, 1H, OCHC*H*H), 1.59 (br. s, 1H, O*H*), 1.50–1.19 (m, 16H, alkyl chain), 0.88 (t, *J* = 6.74 Hz, 3H, C*H*_3_); [α]_D_ = +2.9 (*c* 0.99, CH_2_Cl_2_); These data are in agreement with those previously reported by Zhu *et al*. [13].

#### 3.2.2. (3*R*)-3-Hydroxydodecanoic Acid (**5**)

To a solution of homoallylic alcohol **16** (2.10 g, 10.6 mmol) in acetone/water (8:1 v/v, 100 mL) was added OsO_4_ (0.13 mL, 11.0 μmol, 2.5 wt% in *t*-BuOH) and *N*-methylmorpholine-*N*-oxide (2.48 g, 21.2 mmol). The mixture was left to stir at room temperature for 16 h after which time OsO_4_ (0.1 mL, 8.00 μmol, 2.5 wt% in *t*-BuOH) was added and it was left to stir for another 16 h. The reaction mixture was poured onto water (150 mL) and extracted with EtOAc (3 × 100 mL). The combined organic layers were washed with water (70 mL) and saturated aqueous NaCl (70 mL), dried (MgSO_4_), filtered and concentrated *in vacuo*. The crude solid was used without further purification. To a solution of the crude product in THF/water (9:1.25 v/v, 103 mL) was added sodium periodate (3.40 g, 15.9 mmol). The mixutre was left to stir at room temperature for 2 h. The reaction mixture was then poured onto water (150 mL) and extracted with EtOAc (3 × 100 mL). The combined organic layers were washed with saturated aqueous NaHCO_3_ (70 mL), dried (MgSO_4_), filtered and concentrated *in vacuo*. The crude oil was used without further purification. To a solution of the crude product in *t*-BuOH/water (1:1 v/v, 100 mL) was added sodium chlorite (4.80 g, 53 mmol) and sodium dihydrogen phosphate (6.35 g, 53 mmol). The mixture was left to stir at room temperature for 16 h. The reaction mixture was then poured onto water (150 mL) which was acidified to pH = 1 with aqueous 1 M HCl. This was then extracted with EtOAc (3 × 100 mL). The combined organic layers were washed with saturated aqueous NaCl (70 mL), dried (MgSO_4_), filtered and concentrated *in vacuo*. The crude product with purified by flash chromatography (eluent: 1:99:0.1 v/v/v→3.5:96.5:0.1 v/v/v MeOH/CH_2_Cl_2_/AcOH) to yield acid **5** as a white solid (1.77 g, 77%).

The purified acid **5** (1.70 g, 7.89 mmol) was dissolved in MeCN (56 mL) and dicyclohexylamine (1.72 mL, 8.68 mmol) was added. The mixture was then recrystallized from MeCN three times. The salt was then dissolved in water (40 mL) and aqueous 1 M HCl was added until the solution reached pH = 1. This solution was extracted with Et_2_O (5 × 40 mL). The combined organic layers were dried (Na_2_SO_4_), filtered and concentrated *in vacuo* to yield acid **5** as a white solid (1.34 g, 75%). 

^1^H-NMR (CDCl_3_, 400 MHz) δ (ppm) 4.08–3.99 (m, 1H, HOC*H*), 2.58 (dd, *J* = 3.1, 16.5 Hz, 1H, CH*H*COOH), 2.48 (dd, *J* = 8.9, 17.0 Hz, 1H, C*H*HCOOH), 1.61–1.18 (m, 16H, alkyl chain), 0.88 (t, *J =* 6.5 Hz, 3H, C*H*_3_); [α]_D_ = −21.0 (*c* 0.11, CH_2_Cl_2_); LRMS (−ESI) *m/z* 215 [M − H]^–^; mp. 62.3 °C. These data are in agreement with those previously reported by Guaragna *et al*. [11].

### 3.3. Mosher’s Ester Determination of Enantiomeric Excess

#### 3.3.1. Methyl (3*R*)-3-Hydroxydodecanoate (Methyl Ester-**5**)

To a solution of acid **5** (50 mg, 0.23 mmol) in MeOH (1 mL) at 0 °C was added thionyl chloride (84 μL, 1.16 mmol) dropwise. The solution was stirred at room temperature for 16 h. The reaction mixture was concentrated *in vacuo* and the crude product was purified by flash chromatography (eluent: 20:80 v/v EtOAc/petroleum ether) to yield the methyl ester of **5** as a white solid (47 mg, 89%).

^1^H-NMR (CDCl_3_, 300 MHz) δ (ppm) 4.05–3.95 (m, 1H, C*H*OH), 3.71 (s, 3H, OC*H*_3_), 2.52 (dd, *J* = 3.3, 16.3, 1H, CH*H*COOMe), 2.40 (dd, *J* = 8.9, 16.6 Hz, 1H, C*H*HCOOMe), 1.58–1.20 (m, 16H, alkyl chain), 0.87 (t, *J* = 6.4 Hz, 3H, C*H*_3_); [α]_D_ = −9.6 (*c* 0.37 , CH_2_Cl_2_); HRMS (+ESI) Calc. for C_13_H_26_O_3_Na [M + Na]^+^: 253.1774, found: 253.1775; mp. 28.5 °C. These data are in agreement with those previously reported by Hasdemir and co-workers [19].

#### 3.3.2. Tridec-1-en-4-ol (*rac*-**16**)

To a solution of distilled decanal (**17**) (1.20 mL, 6.40 mmol) in Et_2_O (11.5 mL) at −42 °C was added allyl magnesium bromide (7.68 mL, 7.68 mmol, 1 M in Et_2_O) dropwise. The mixture was left to stir at this temperature before being warmed to room temperature for 1 h. The reaction mixture was quenched at 0 °C with saturated aqueous NH_4_Cl (5 mL), poured onto saturated aqueous NH_4_Cl (40 mL) and extracted with Et_2_O (3 × 40 mL). The combined organic layers were washed with saturated aqueous NaHCO_3_ (30 mL) and water (30 mL), dried (Na_2_SO_4_), filtered and concentrated *in vacuo* to yield homoallylic alcohol *rac-***16** as a colourless oil (1.26 g, 99%).

^1^H-NMR (CDCl_3_, 300 MHz) δ (ppm) 5.92–5.75 (m, 1H, OCCH_2_C*H*CH_2_), 5.19–5.13 (m, 1H, OCCH_2_CHCH*H*), 5.13–5.09 (m, 1H, OCCH_2_CHC*H*H), 3.70–3.59 (m, 1H, C*H*OH), 2.37–2.25 (m, 1H, OCHCH*H*), 2.20–2.08 (m, 1H, HOCHC*H*H), 1.52–1.20 (m, 16H, alkyl chain), 0.88 (t, *J* = 6.36 Hz, 3H, C*H*_3_); LRMS (+APCI) *m*/*z* 181 [M − OH]^+^. These data are in agreement with those previously reported by Dommisse *et al*. [20].

#### 3.3.3. 3-Hydroxydodecanoic Acid (*rac*-**5**)

To a solution of homoallylic alcohol *rac*-**16** (217 mg, 1.10 mmol) in acetone/water (8:1 v/v, 15 mL) was added OsO_4_ (0.69 mL, 0.055 mmol, 2.5 wt% in *t*-BuOH) and *N*-methylmorpholine-*N*-oxide (257 mg, 2.19 mmol). The mixture was left to stir at room temperature for 16 h. The reaction mixture was poured onto water (15 mL) and extracted with EtOAc (3 × 20 mL). The combined organic layers were washed with water (20 mL) and saturated aqueous NaCl (20 mL), dried (MgSO_4_), filtered and concentrated *in vacuo*. The crude solid was used without further purification. To a solution of the crude product in THF/water (9:1.25 v/v, 10.25 mL) was added sodium periodate (351 mg, 1.64 mmol). The mixutre was stirred at room temperature for 16 h. The reaction mixture was then poured onto water (15 mL) and extracted with EtOAc (3 × 20 mL). The combined organic layers were washed with saturated aqueous NaCl (20 mL), dried (MgSO_4_), filtered and concentrated *in vacuo*. The crude oil was used without further purification. To a solution of the crude product in *t*-BuOH/water (1:1 v/v, 12 mL) was added sodium chlorite (992 mg, 11.0 mmol) and sodium dihydrogen phosphate (1.31 g, 11.0 mmol). The mixture was stirred at room temperature for 16 h. The reaction mixture was then poured onto water (15 mL) which was acidified to pH = 2 with aqueous 1 M HCl. This was then extracted with EtOAc (3 × 20 mL). The combined organic layers were washed with saturated aqueous NaCl (1 × 20 mL), dried (MgSO_4_), filtered and concentrated *in vacuo*. The crude product with purified by flash chromatography (eluent: 3:97:0.1 v/v/v→5:95:0.1 v/v/v MeOH/CH_2_Cl_2_/AcOH) to yield acid *rac*-**5** as a white solid (142 mg, 60%).

^1^H-NMR (CDCl_3_, 400 MHz) δ (ppm) 4.07–3.99 (m, 1H, HOC*H*), 2.57 (dd, *J* = 3.1, 16.6 Hz, 1H, CH*H*COOH), 2.47 (dd, *J* = 9.0, 16.4 Hz, 1H, C*H*HCOOH), 1.61–1.19 (m, 16H, alkyl chain), 0.88 (t, *J =* 6.7 Hz, 3H, C*H*_3_); LRMS (−ESI) *m/z* 215 [M − H]^−^; mp. 69.0 °C. These data are in agreement with those previously reported by Skogh and Guaragna *et al*. [11,21].

#### 3.3.4. Methyl 3-Hydroxydodecanoate (*rac*-Methyl Ester **5**)

To a solution of acid *rac*-**5** (50 mg, 0.231 mmol) in MeOH (1 mL) at 0 °C was added thionyl chloride (84 μL, 1.16 mmol) dropwise. The solution was stirred at room temperature for 16 h. The reaction mixture was concentrated *in vacuo* and the crude product was purified by flash chromatography (eluent: 20:80 v/v EtOAc/petroleum ether) to yield the methyl ester of *rac*-**5** as a colourless oil (48 mg, 90%). 

^1^H-NMR (CDCl_3_, 300 MHz) δ (ppm) 4.05–3.94 (m, 1H, C*H*OH), 3.71 (s, 3H, OC*H*_3_), 2.52 (dd, *J* = 3.3, 16.5, 1H, CH*H*COOMe), 2.40 (dd, *J* = 8.9, 16.7 Hz, 1H, C*H*HCOOMe), 1.59–1.2 (m, 16H, alkyl chain), 0.87 (t, *J* = 6.4 Hz, 3H, C*H*_3_); HRMS (+ESI) Calc. for C_13_H_26_O_3_Na [M + Na]^+^: 253.1774, found: 253.1774. These data are in agreement with those previously reported by Hasdemir and co-workers [19].

#### 3.3.5. General Procedure for Mosher’s Esterification

To a solution of alcohol methyl ester-**5**, *rac*-methyl ester-**5**, 16 or *rac*-**16**, (1 equiv., ~5–10 mg) in CH_2_Cl_2_ (0.025 M) was added MTPA (2 equiv.), DIC (3 equiv.) and DMAP (1.5 equiv.). The solution was stirred at room temperature for 16 h and analysed by TLC. If starting material was present then additional α-methoxy-α-trifluoromethylphenylacetic acid (MTPA) (1 equiv.), DIC (1 equiv.) and DMAP (1 equiv.) were added and the reaction was left to stir at room temperature until complete consumption of starting material, and more reagents were added if necessary. The reaction mixture was then poured onto water (5 mL) and extracted with CH_2_Cl_2_ (3 × 4 mL). The combined organic layers were washed with aqueous citric acid (5 mL, 10% wt/v) and saturated aqueous NaHCO_3_ (5 mL), dried (MgSO_4_), filtered and concentrated *in vacuo*. The crude product was then analysed by ^1^H-NMR and ^19^F-NMR to determine the *ee* (See Supplementary Information) [14].

### 3.4. Solid Phase Peptide Synthesis (0.1 mmol)

Loading: Weinreb amide resin (139 mg, 0.1 mmol) was swollen in DMF (3 mL) for 5 min. The resin was then shaken in a solution of piperdine/DMF (3 mL, 1:9 v/v) for 3 min and this procedure repeated. The resin was filtered and washed with DMF (5 × 3 mL), DCM (5 × 3 mL) and DMF (5 × 3 mL). A solution of Fmoc-Leu-OH (177 mg, 0.5 mmol) or Fmoc-Val-OH (167 mg, 0.5 mmol), HATU (190 mg, 0.5 mmol) and Hünig’s base (170 μL, 1 mmol) in DMF (1 mL) was added to the resin and shaken at room temperature for 6 h. The resin was filtered and washed with DMF (5 × 3 mL), CH_2_Cl_2_ (5 × 3 mL) and DMF (5 × 3 mL). The procedure was repeated.

Fmoc-deprotection: The resin was then shaken in a solution of piperdine/DMF (3 mL, 1:9 v/v) for 3 min and the procedure repeated. The resin was filtered and washed with DMF (5 × 3 mL), CH_2_Cl_2_ (5 × 3 mL) and DMF (5 × 3 mL).

Fmoc-Gln(Trt)-OH, Fmoc-Asn(Trt)-OH and lauric acid (**10**) coupling: A solution of acid (0.4 mmol), PyBOP (208 mg, 0.4 mmol) and NMM (88 μL, 0.8 mmol) in DMF (1 mL) was added to the resin and shaken at room temperature for 2 h. The resin was filtered and washed with DMF (5 × 3 mL), CH_2_Cl_2_ (5 × 3 mL) and DMF (5 × 3 mL).

(3*R*)-hydroxy lauric acid (**5**) and (3*R*)-hydroxy myristic acid (**11**) coupling: A solution of acid (0.12 mmol), HATU (45 mg, 0.12 mmol) and Hünig’s base (42 μL, 2.4 mmol) in DMF (1 mL) was added to the resin and shaken at room temperature for 16 h. The resin was filtered and washed with DMF (5 × 3 mL), CH_2_Cl_2_ (5 × 3 mL) and DMF (5 × 3 mL).

Capping: The resin was treated with a solution of pyridine/Ac_2_O (3 mL, 9:1 v/v) for 2 min. The resin was filtered and washed with DMF (5 × 3 mL), CH_2_Cl_2_ (5 × 3 mL) and DMF (5 × 3 mL).

Side-chain deprotection: The resin was treated with a solution of TFA:TIS:H_2_O (9:0.5:0.5, v/v/v) (3 mL) and shaken for 1 h. The resin was then washed with CH_2_Cl_2_ (20 × 3 mL) and then dried thoroughly under vacuum.

### 3.5. Resin Cleavage Conditions (0.1 mmol)

The resin bound peptide (0.1 mmol) was swollen in dry THF (7.5 mL) in a round bottom flask for 1 h. To this mixture at 0 °C was added a solution of LiAlH_4_ (0.5 mL, 1 mmol, 2 M in THF). The mixture was stirred at this temperature for 40 min, before diluting with THF (5 mL) and quenching with saturated aqueous KHSO_4_ solution (2.25 mL) and saturated aqueous NaKTartrate solution (1.5 mL). The solution was then warmed to room temperature over 40 min. The supernatant was decanted before CH_2_Cl_2_ (30 mL) was added to the resultant solid and filtered through Celite. The filtrate was combined with the supernatant liquid, and a white solid precipitated. Water (50 mL) was added and the solution was extracted with further CH_2_Cl_2_ (100 mL). The combined organic extracts were then dried (MgSO_4_), filtered and concentrated *in vacuo* to yield the desired peptide. 

### 3.6. Synthesis of Peptides ***2*** and ***6**–**8***

#### 3.6.1. Fellutamide B (**2**)

Weinreb amide resin (115 μmol) was loaded with Fmoc-Leu-OH, and then Fmoc-Gln(Trt)-OH, Fmoc-Asn(Trt)-OH and (3*R*)-hydroxy lauric acid (**5**) were coupled sequentially according to the SPPS protocol. Cleavage from the resin as detailed in Section 3.5, yielded fellutamide B (**2**) as a white solid (35 mg, 55%). 

^1^H-NMR (*d*_6_-DMSO, 500 MHz) δ (ppm) 9.35 (s, 1H, H-1), 8.26 (d, *J* = 7.5 Hz, 1H, H-6), 8.15–8.08 (m, 2H, H-13 + H-19), 7.43 (s, 1H, H-18), 7.21 (s, 1H, H-12), 6.92 (s, 1H, H-18), 6.75 (s, 1H, H-12), 4.64 (d, *J* = 4.7 Hz, 1H, O*H*), 4.53–4.44 (m, 1H, H-15), 4.21–4.15 (m, 1H, H-8), 4.07–4.00 (m, 1H, H-2), 3.78 (br s, 1H, H-22), 2.55 (dd, *J* = 6.6, 15.3, 1H, H-16), 2.45 (dd, *J* = 7.0, 15.3 Hz, 1H, H-16), 2.27–2.15 (m, 2H, 2 × H-21), 2.15–2.03 (m, 2H, 2 × H-10), 2.01–1.86 (m, 1H, H-9), 1.81–1.70 (m, 1H, H-9), 1.67–1.58 (m, 1H, H-4), 1.56–1.45 (m, 2H, 2 × H-3), 1.42–1.17 (m, 16H, H-23-H-30), 0.93–0.75 (m, 9H, 3 × H-5+3 × H-5′ + 3 × H-31); ^13^C-NMR (*d*_6_-DMSO, 125 MHz) δ (ppm) 201.6, 173.8, 171.9, 171.8, 171.2, 171.0, 67.5, 56.6, 52.4, 49.9, 43.3, 37.0, 36.9, 31.4, 31.3, 29.1, 29.0, 28.7, 25.1, 23.9, 23.0, 22.1, 21.3, 13.9, (3 signals obscured); [α]_D_ = −9.3 (*c* 0.17, MeOH); LRMS (+ESI) 578 *m/z* [M + Na]^+^; HRMS (+ESI) Calc. for C_28_H_53_N_5_O_8_Na [M + CH_3_OH + Na]^+^: 610.3786, found: 610.3785; IR ν_max_ (ATR): 3338 (O–H), 1655 (C=O), 1090; mp. decomposition. These data are in agreement with those previously reported by Shigemori *et al.* and Schneekloth Jr. *et al*. [1,2].

#### 3.6.2. Deoxy-fellutamide B (**6**)

Weinreb amide resin (200 μmol) was loaded with Fmoc-Leu-OH and then Fmoc-Gln(Trt)-OH, Fmoc-Asn(Trt)-OH and lauric acid (**10**) were coupled sequentially according to the SPPS protocol. Cleavage from the resin as detailed in Section 3.5, yielded the title compound **6** as a white solid (48 mg, 44% crude). A portion (11 mg) was purified by reverse phase HPLC to yield deoxy-fellutamide B (**6**) as a white solid (6 mg, 55%).

^1^H-NMR (*d*_6_-DMSO, 500 MHz) δ (ppm) 9.35 (s, 1H, H-1), 8.29 (d, *J* = 7.3 Hz, 1H, H-6), 8.10 (d, *J* = 7.6, 1H, H-13), 8.03 (d, *J* = 7.4, 1H, H-19), 7.40 (s, 1H, H-18), 7.20 (s, 1H, H-12), 6.91 (s, 1H, H-18), 6.74 (s, 1H, H-12), 4.49 (app. q, *J* = 7, 1H, H-15), 4.22–4.16 (m, 1H, H-8), 4.07–4.02 (m, 1H, H-2), 2.54 (dd, *J* = 6.5, 15.7 Hz, 1H, H-16), 2.41 (dd, *J* = 6.7, 15.7 Hz, 1H, H-16), 2.15–2.03 (m, 4H, 2 × H-10 + 2 × H-21), 2.00–1.91 (m, 1H, H-9), 1.82–1.71 (m, 1H, H-9), 1.68–1.59 (m, 1H, H-4), 1.53–1.42 (m, 4H, 2 × H-3 + 2 × H-22), 1.32–1.18 (m, 16H, H-23-H-30), 0.90–0.82 (m, 9H, 3 × H-5 + 3 × H5′ + 3 × H-31); ^13^C-NMR (*d*_6_-DMSO, 100 MHz) δ (ppm) 201.6, 173.7, 172.5, 171.8, 171.7, 171.2, 56.6, 52.4, 49.8, 37.1, 36.3, 35.1, 31.3, 29.0, 29.0, 28.9, 28.8, 28.7, 28.7, 27.4, 25.1, 23.9, 23.1, 22.1, 21.3, 13.9, (1 signal obscured); [α]_D_ = −23.7 (*c* 0.14, MeOH); LRMS (+ESI) 562 *m/z* [M + Na]^+^; HRMS (+ESI) Calc. for C_27_H_49_N_5_O_6_Na [M + Na]^+^: 562.3575, found: 562.3564; IR ν_max_ (ATR): 2925, 1742 (C=O), 1627 (C=O), 1547, 1365, 1216; mp. decomposition

#### 3.6.3. Deoxy-fellutamide C (**7**)

Weinreb amide resin (100 μmol) was loaded with Fmoc-Val-OH and then Fmoc-Gln(Trt)-OH, Fmoc-Asn(Trt)-OH and (3*R*)-hydroxy myristic acid (**11**) were coupled sequentially according to the SPPS protocol. Cleavage from the resin as detailed in Section 3.5, yielded deoxy-fellutamide C (**7**) as a white solid (30 mg, 53%). 

^1^H-NMR (*d*_6_-DMSO, 500 MHz) δ (ppm) 9.40 (d, *J* = 1.3 Hz, 1H, H-1), 8.14 (d, *J* = 7.6, 1H, H-6), 8.09 (d, *J* = 7.8 Hz, 1H, H-19), 8.07 (d, *J* = 7.8 Hz, 1H, H-13), 7.40 (s, 1H, H-18), 7.23 (s, 1H, H-12), 6.91 (s, 1H, H-18), 6.76 (s, 1H, H-12), 4.65 (d, *J* = 4.9 Hz, 1H, O*H*), 4.55–4.47 (m, 1H, H-15), 4.27–4.22 (m, 1H, H-8), 3.98 (ddd, *J* = 1.3, 5.7, 7.9 Hz, 1H, H-2), 3.81–3.73 (m, 1H, H-22), 2.55 (dd, *J* = 6.4, 15.0 Hz, 1H, H-16), 2.44 (dd, *J* = 7.0, 15.4 Hz, 1H, H-16), 2.24–2.05 (m, 5H, 2 × H-21 + 2 × H-10 + H-3), 2.02–1.90 (m, 1H, H-9), 1.82–1.72 (m, 1H, H-9), 1.39–1.16 (m, 20H, H-23-H-32), 0.90 (d, *J* = 6.9 Hz, 3H, 3 × H-4), 0.87–0.83 (m, 6H, 3 × H-5 + 33 × H-33) ^13^C-NMR (*d*_6_-DMSO, 125 MHz) δ (ppm) 201.4, 173.9, 171.9, 171.8, 171.2, 171.1, 67.5, 63.1, 52.4, 49.8, 43.4, 37.0, 36.9, 31.4, 31.3, 29.1, 29.1, 29.0, 29.0, 28.7, 25.1, 22.1, 19.0, 18.0, 13.9, (3 signals obscured); [α]_D_ = −13.5 (*c* 0.12, MeOH); LRMS (+ESI) 592 *m/z* [M + Na]^+^; HRMS (+ESI) Calc. for C_28_H_51_N_5_O_7_Na [M + Na]^+^: 592.3681, found: 592.3678; IR ν_max_ (ATR): 3332 (O–H), 1654 (C=O), 1083; mp. decomposition.

#### 3.6.4. Deoxy-fellutamide D (**8**)

Weinreb amide resin (100 μmol) was loaded with Fmoc-Leu-OH and then Fmoc-Gln(Trt)-OH, Fmoc-Asn(Trt)-OH (48) and (3*R*)-hydroxy myristic acid (**11**) were coupled sequentially according to the SPPS protocol. Cleavage from the resin as detailed in Section 3.5, yielded deoxy-fellutamide D (**8**) as a white solid (35 mg, 60%). 

^1^H-NMR (*d*_6_-DMSO, 500 MHz) δ (ppm) 9.35 (s, 1H, H-1), 8.26 (d, *J* = 7.6 Hz, 1H, H-6), 8.10 (app. dd, *J* = 1.7, 7.3 Hz, 2H, H-13 + H-19), 7.42 (s, 1H, H-18), 7.21 (s, 1H, H-12), 6.92 (s, 1H, H-18), 6.75 (s, 1H, H-12), 4.64 (d, *J* = 5.1 Hz, 1H, O*H*), 4.53–4.46 (m, 1H, H-15), 4.22–4.16 (m, 1H, H-8), 4.06–4.01 (m, 1H, H-2), 3.82–3.73 (m, 1H, H-22), 2.56 (dd, *J* = 6.7, 15.7 Hz, 1H, H-16), 2.45 (dd, *J* = 7.0, 15.3 Hz, 1H, H-16), 2.26–2.16 (m, 2H, 2 × H-21), 2.14–2.03 (m, 2H, 2 × H-10), 2.01–1.91 (m, 1H, H-9), 1.81–1.69 (m, 1H, H-9), 1.68–1.57 (m, 1H, H-4), 1.51–1.45 (m, 2H, 2 × H-3), 1.39–1.16 (m, 20H, H-23-H-32), 0.91–0.76 (m, 9H, 3 × H-5 + 3 × H-5′+3 × H-33) ^13^C-NMR (*d*_6_-DMSO, 125 MHz) δ (ppm) 201.6, 173.8, 171.9, 171.8, 171.2, 171.1, 67.5, 56.6, 52.4, 49.8, 43.4, 36.9, 36.3, 31.4, 31.3, 29.1, 29.1, 29.0, 28.7, 25.12, 23.9, 23.1, 22.1, 21.3, 13.9, (4 signals obscured); [α]_D_ = −15.0 (*c* 0.13, MeOH); LRMS (+ESI) 606 *m/z* [M + Na]^+^; HRMS (+ESI) Calc. for C_29_H_53_N_5_O_7_Na [M + Na]^+^: 606.3837, found: 606.3839; IR ν_max_ (ATR): 3356 (O–H), 1740 (C=O), 1648 (C=O), 1084; mp. decomposition.

### 3.7. Mycobacterium tuberculosis *Inhibition Assays*

*M. tuberculosis* H37Rv (ATCC 27294) was grown in Middlebrook 7H9 broth medium supplemented with ADC (Difco Laboratories, Detroit, MI, USA), 0.5% glycerol, and 0.05% Tween-80. Freshly seeded cultures were grown at 37 °C to mid-exponential phase (OD600 0.4–0.8) for use in resazurin reduction inhibition assay [22,23,24]. *M. tuberculosis* H37Rv, grown to mid-exponential phase, was diluted to OD600 0.002 in 7H9S media (Middlebrook 7H9 with ADC, 0.05% glycerol, 0.05% Tween-80, 1% tryptone) and ~2 × 10^4^ CFU/mL added to the wells of 96-well microtiter plates with serial dilutions of the inhibitors, which were originally dissolved in 100% DMSO. Plates were incubated for 5 days at 37 °C in a humidified incubator prior to the addition of a 0.02% resazurin solution (30 μL) and 20% Tween-80 (12.5 μL) to each well. Sample fluorescence was measured after 48 h on a BMG Labtech Polarstar Omega instrument with an excitation wave-length of 530 nm and emission at 590 nm. Changes in fluorescence relative to positive control wells (H37Rv with no inhibitor) minus negative control wells (no H37Rv) were plotted for determination of MIC_50_ values.

As *M. tuberculosis* proteasome inhibitors were more active in the presence of non-lethal levels of NO [6], *M. tuberculosis* was cultured with the synthetic fellutamides in the presence of DETA-NO, which releases NO with T_1/2_ of ~20 h [17]. *M. tuberculosis* H37Ra (ATCC 25177) was grown to mid-exponential phase (OD600 0.4–0.8) in ADC-supplemented Middlebrook 7H9 medium, and then ~2 × 10^5^ bacilli in 100 μL were incubated with serial dilutions of the inhibitors and 50 μM DETA-NO in 100 μL in 96-well microtiter plates. Plates were incubated for 5 days at 37 °C with the addition of DETA-NO at 24, 48, 72, 96 and 120 h to a final concentration of 50 μM. Absorbance was measured daily on BMG Labtech Polarstar Omega instrument at OD_600_ prior to the addition of the DETA-NO.

## 4. Conclusions

The work described here represents a significant step towards the synthesis of the fellutamide class of natural products and related analogues. The total synthesis of fellutamide B (**2**) and deoxy-fellutamides B (**6**), C (**7**) and D (**8**) was achieved. The synthesis employed a novel Weinreb amide solid-phase peptide synthesis approach whereby the sensitive *C*-terminal aldehyde could be generated in the final step of the synthesis. An improved enantioselective synthesis of (3*R*)-hydroxy lauric acid (**5**) was also described and was successfully incorporated into a resin bound peptide for the total synthesis of fellutamide B (**2**). Despite the fact that fellutamide B and the natural product analogues did not exhibit activity against *M. tuberculosis* *in vitro*, the reported activity of this natural product class against the *M. tuberculosis* proteasome warrants further investigation. Indeed, it is possible that synthetic analogues which are capable of penetrating the cell wall of *M. tuberculosis* to reach the proteasome may serve as lead compounds for the development of new chemotherapies against latent *M. tuberculosis* infections. 

## Supplementary Files

Supplementary File 1 Supplementary Material (PDF, 1369 KB)
