# Supplementary material for: Total Synthesis of Fellutamide B and Deoxy-Fellutamides B, C, and D
Source: Mar Drugs. 2013 Jul 8;11(7):2382–97. doi: 10.3390/md11072382 (PMC3736429; doi:10.3390/md11072382)

Supplementary Material

Contents

Figure S1. NMR spectra of final compounds (**2** and **6–8**). **2**

Figure S2. NMR spectra of Mosher's esters (**methyl ester-5** and ***rac*-methyl ester-5**). **10**

Figure S3. Comparison of NMR data for fellutamide B (**2**). **14**

Figure S4. Raw data for *in vitro* inhibition assays against *Mycobacterium tuberculosis*. **15**

Figure S1. NMR spectra of final compounds (**2** and **6–8**).

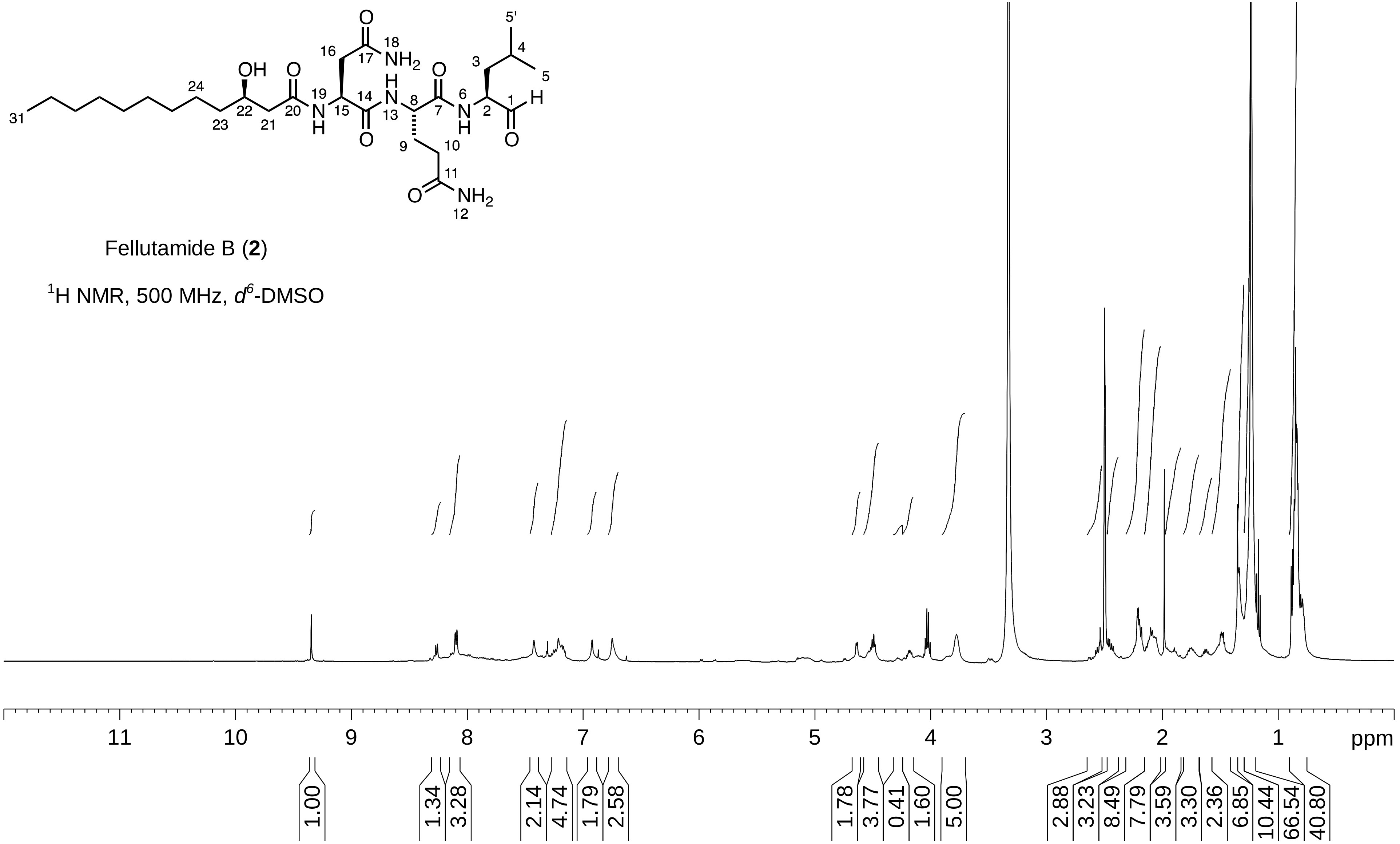

Figure S1. *Cont.*

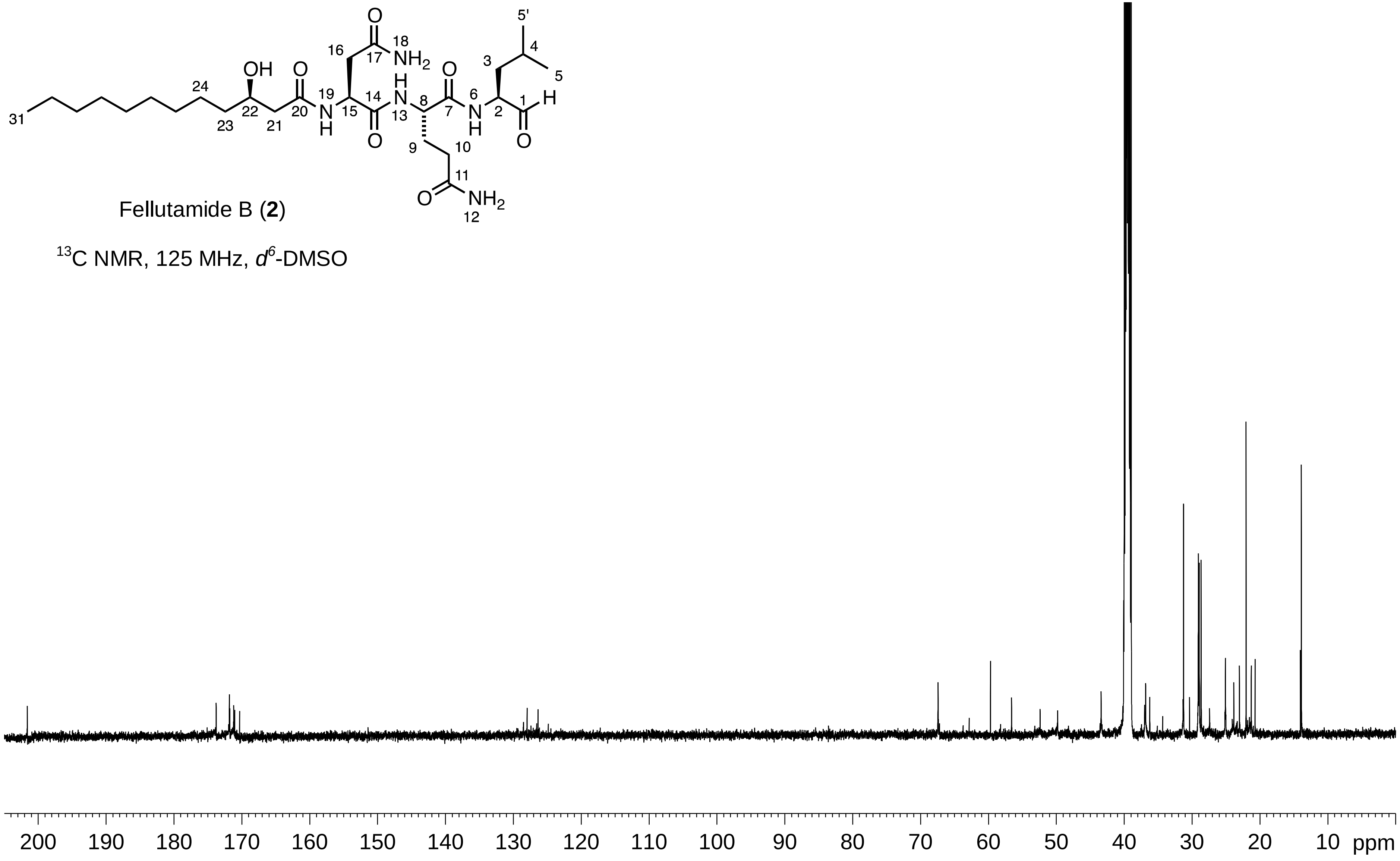

Figure S1. *Cont.*

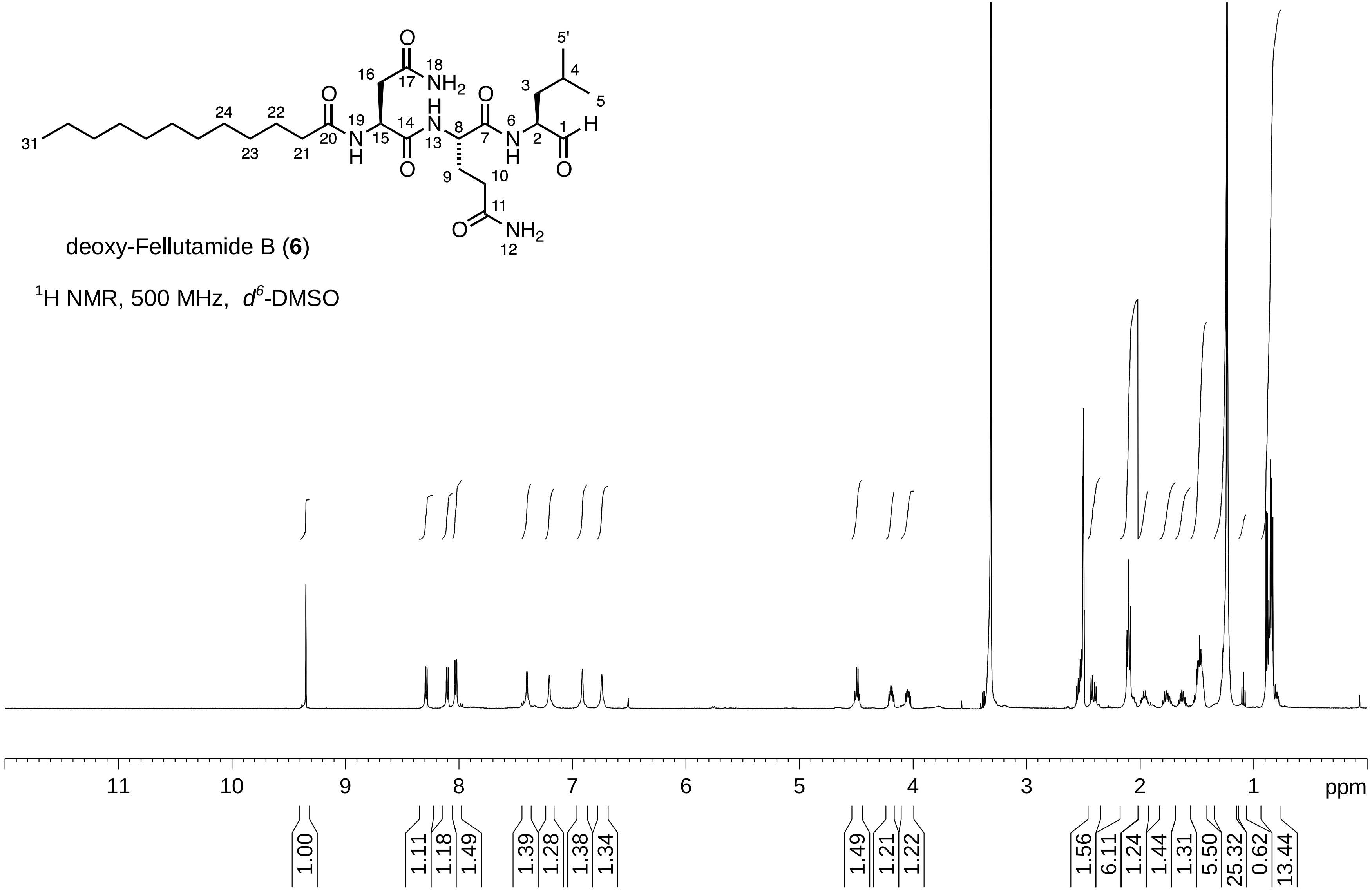

Figure S1. *Cont.*

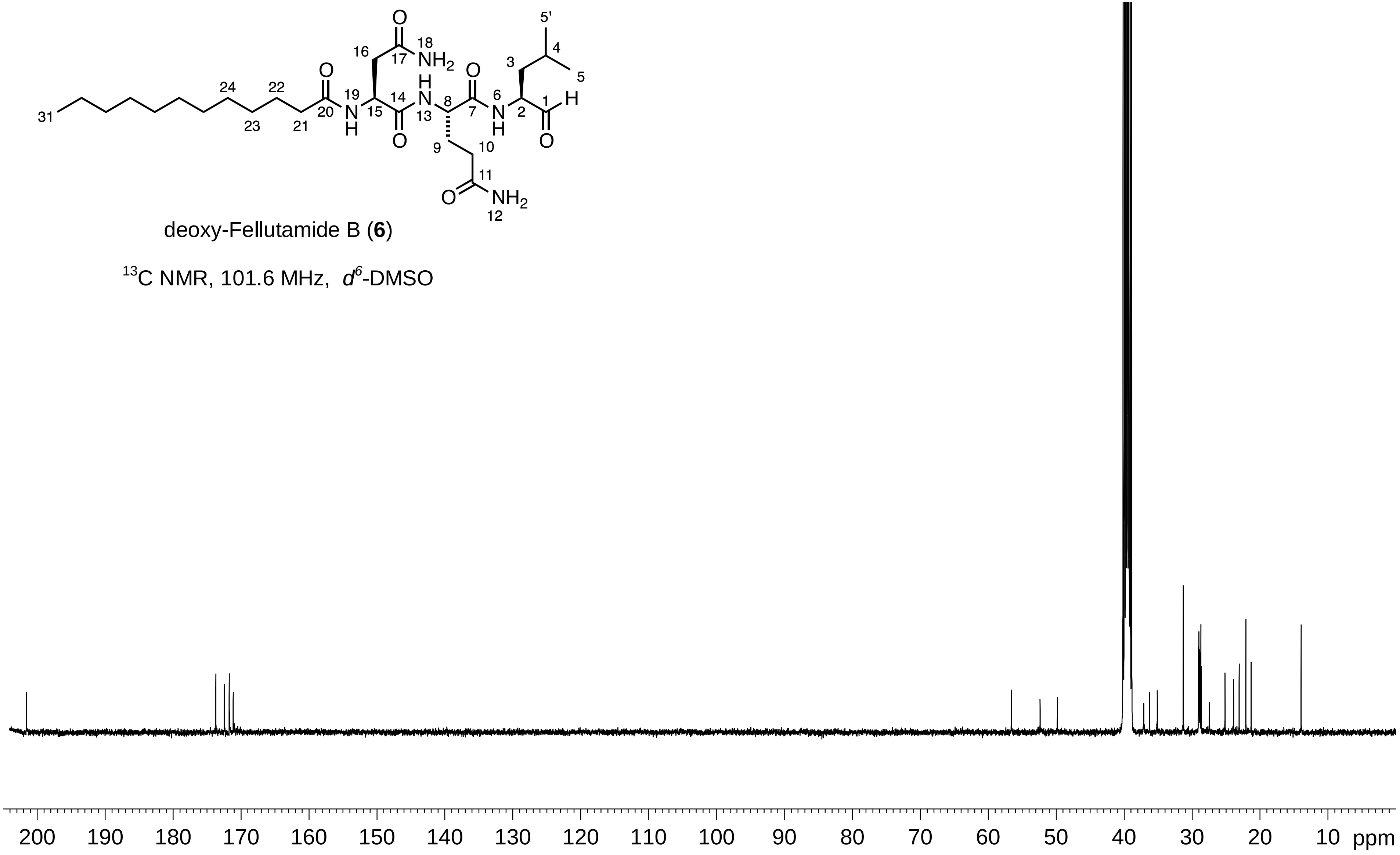

Figure S1. *Cont.*

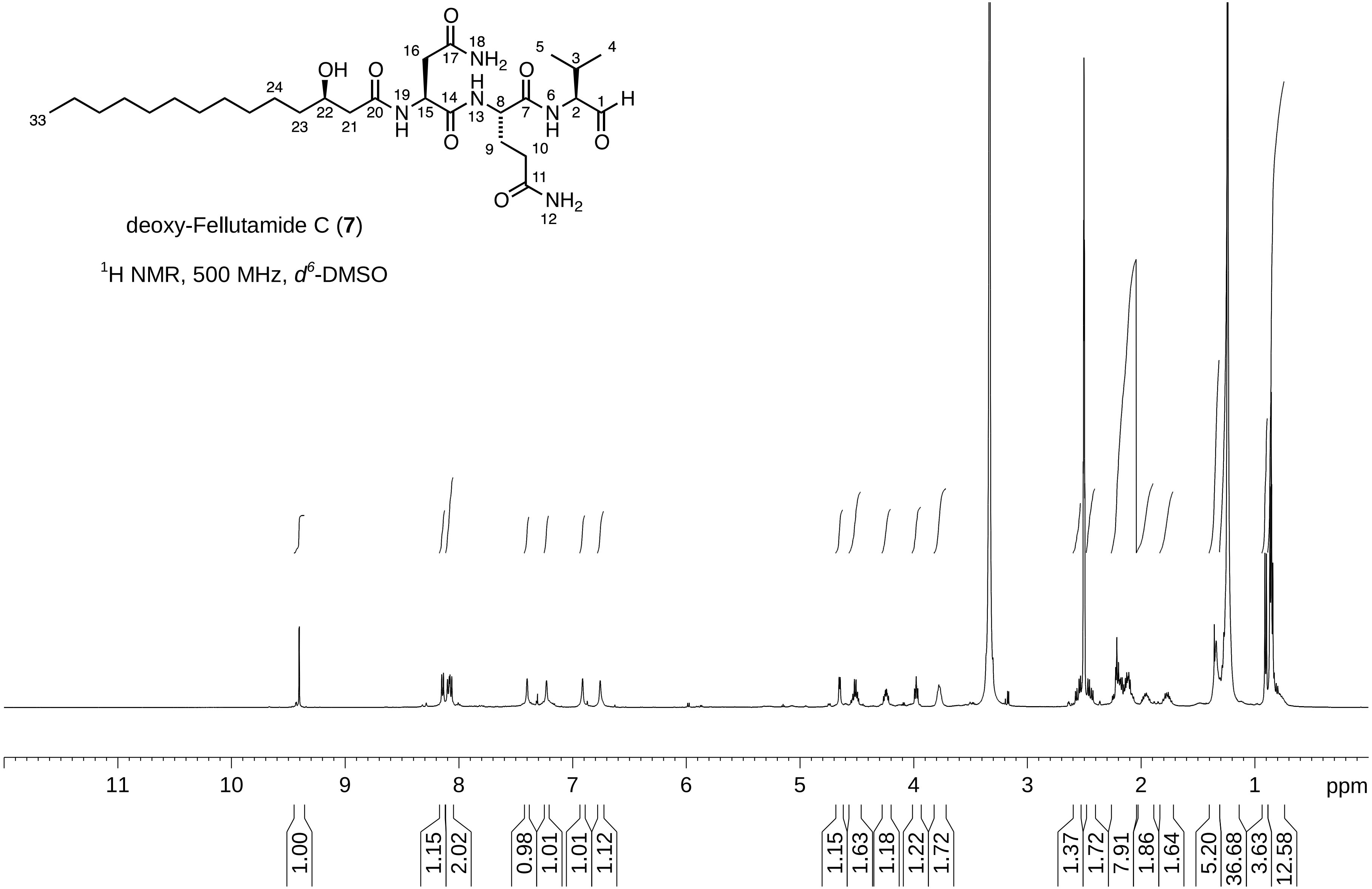

Figure S1. *Cont.*

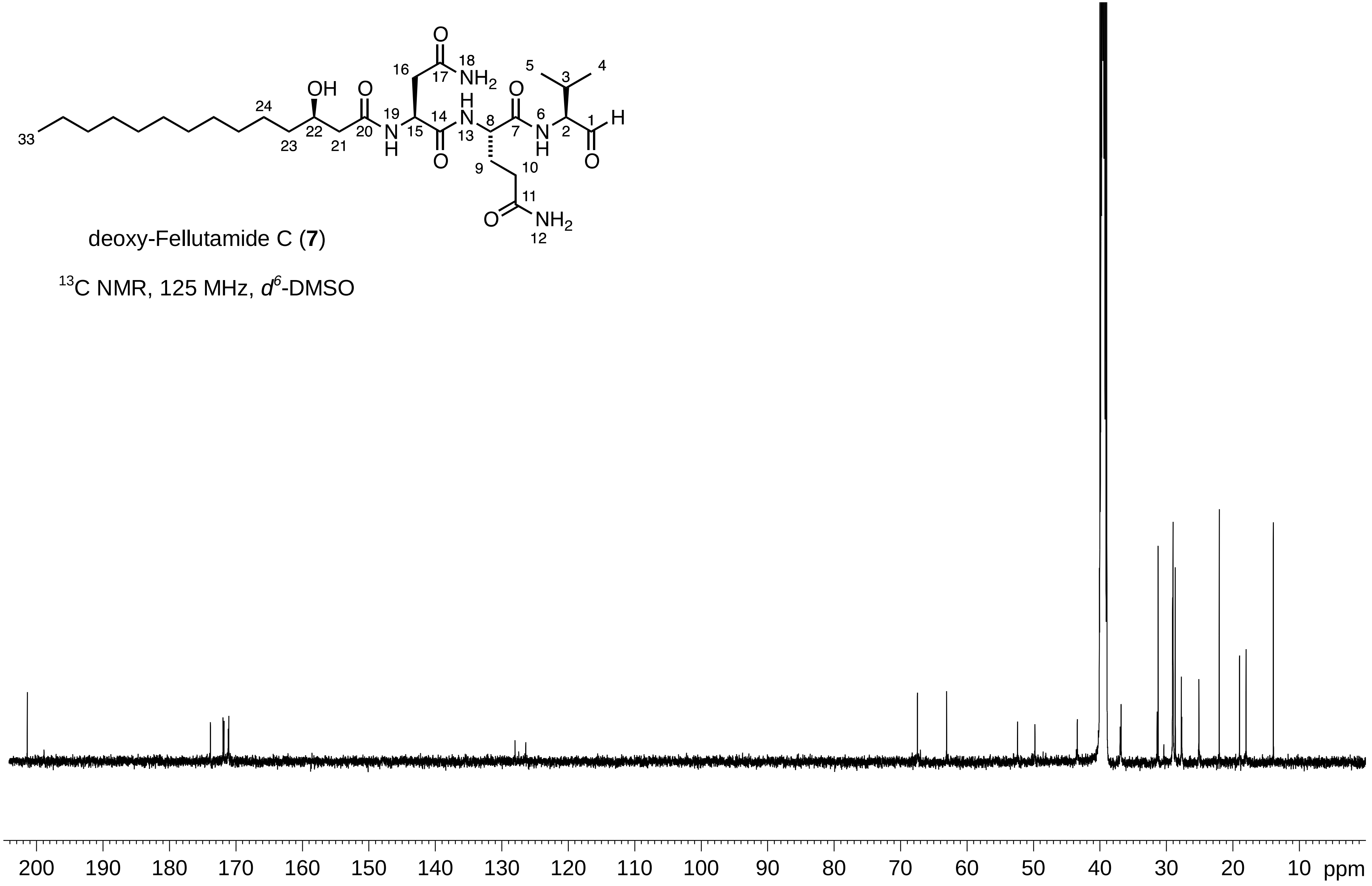

Figure S1. *Cont.*

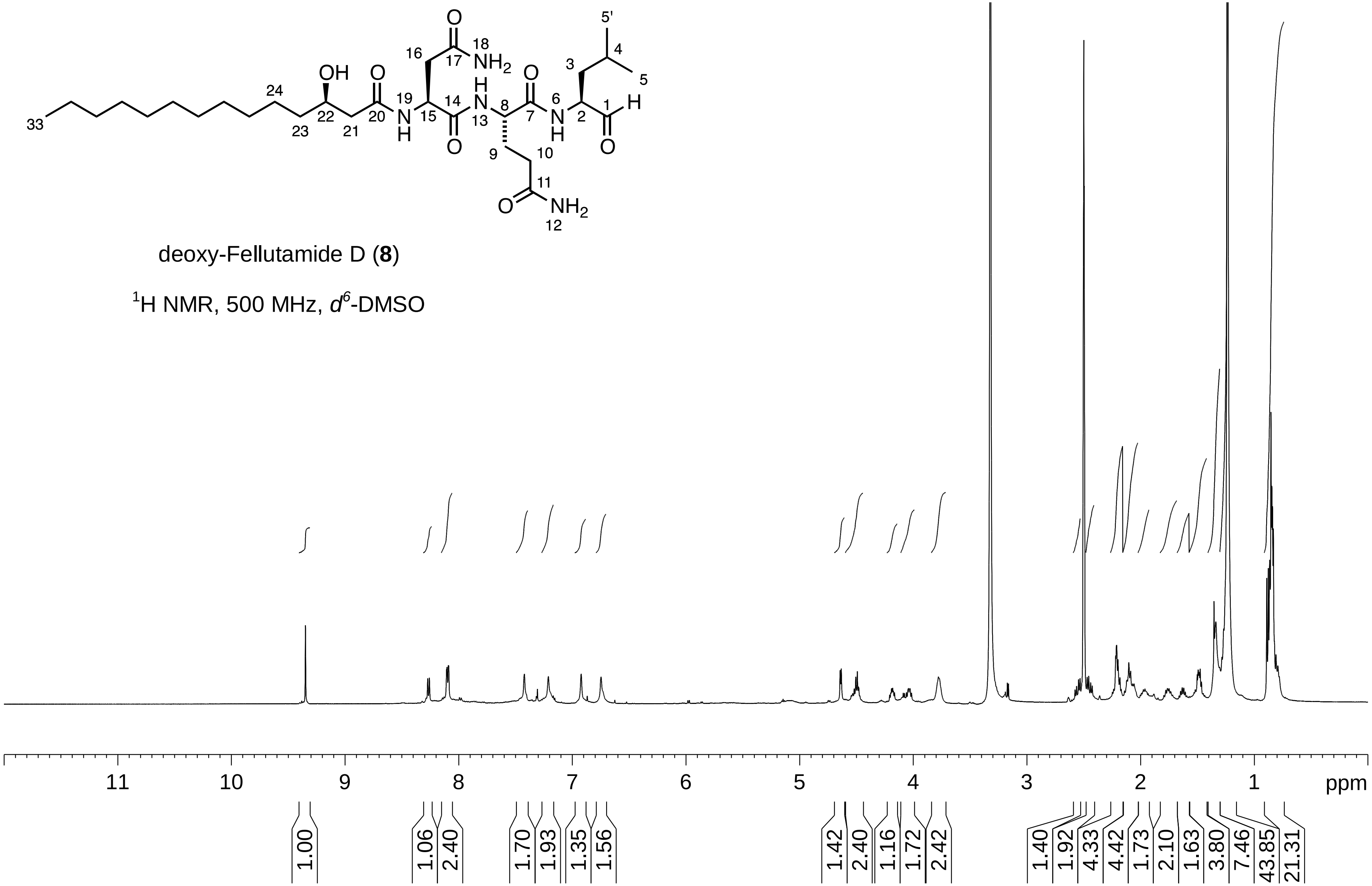

Figure S1. *Cont.*

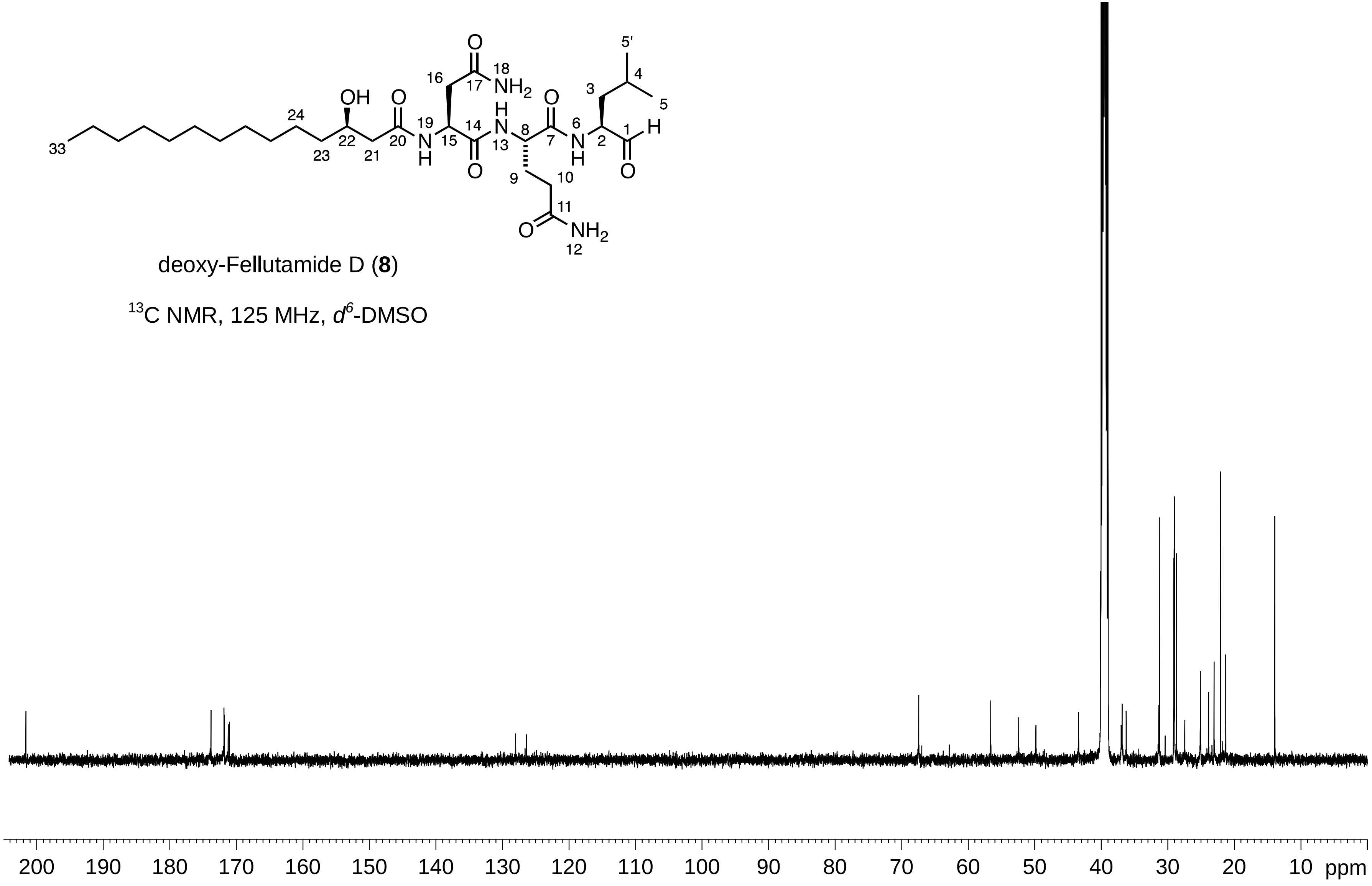

Figure S2. NMR spectra of Mosher's esters (**methyl ester-5** and ***rac*-methyl ester-5**).

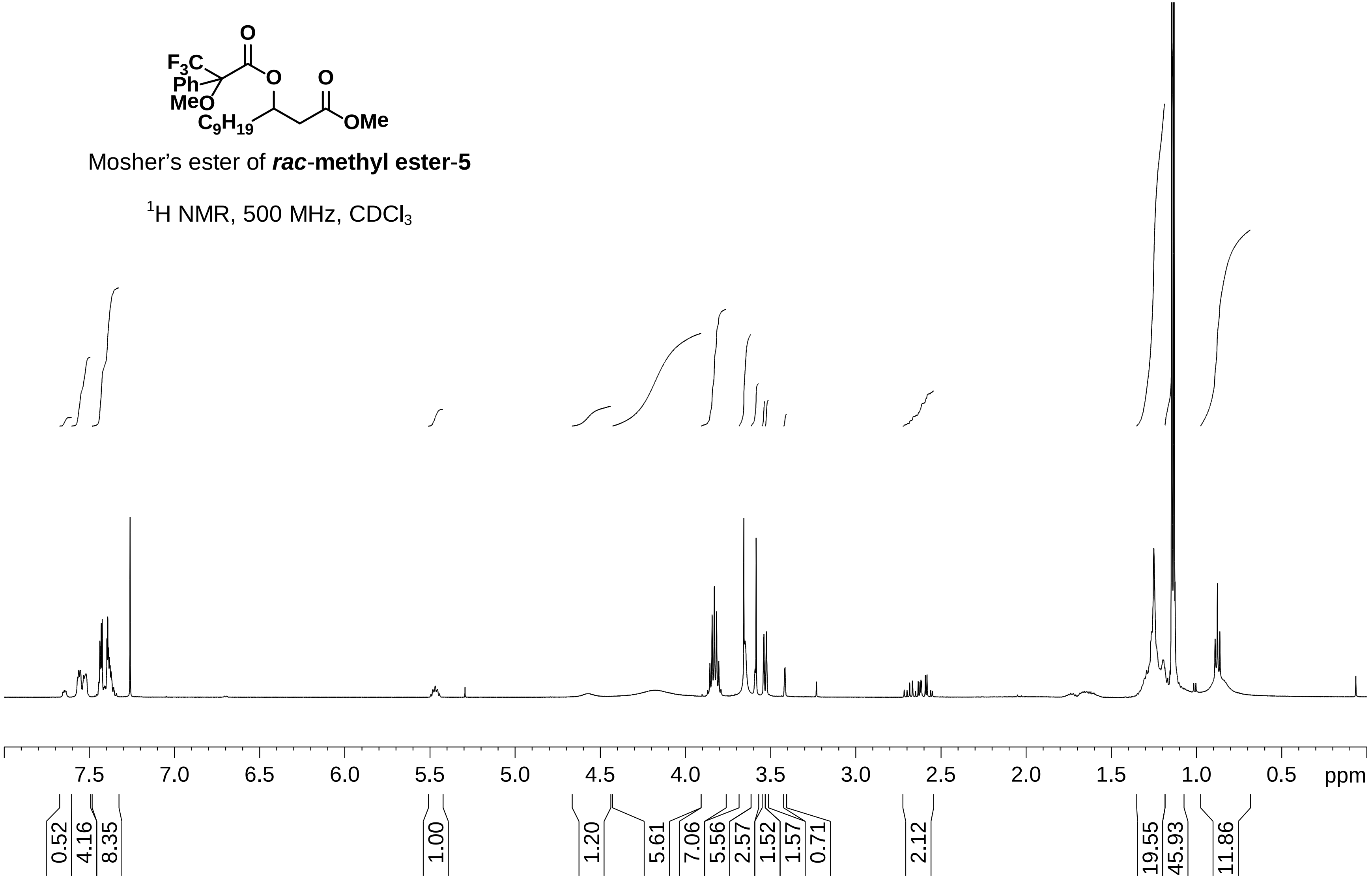

Figure S2. *Cont.*

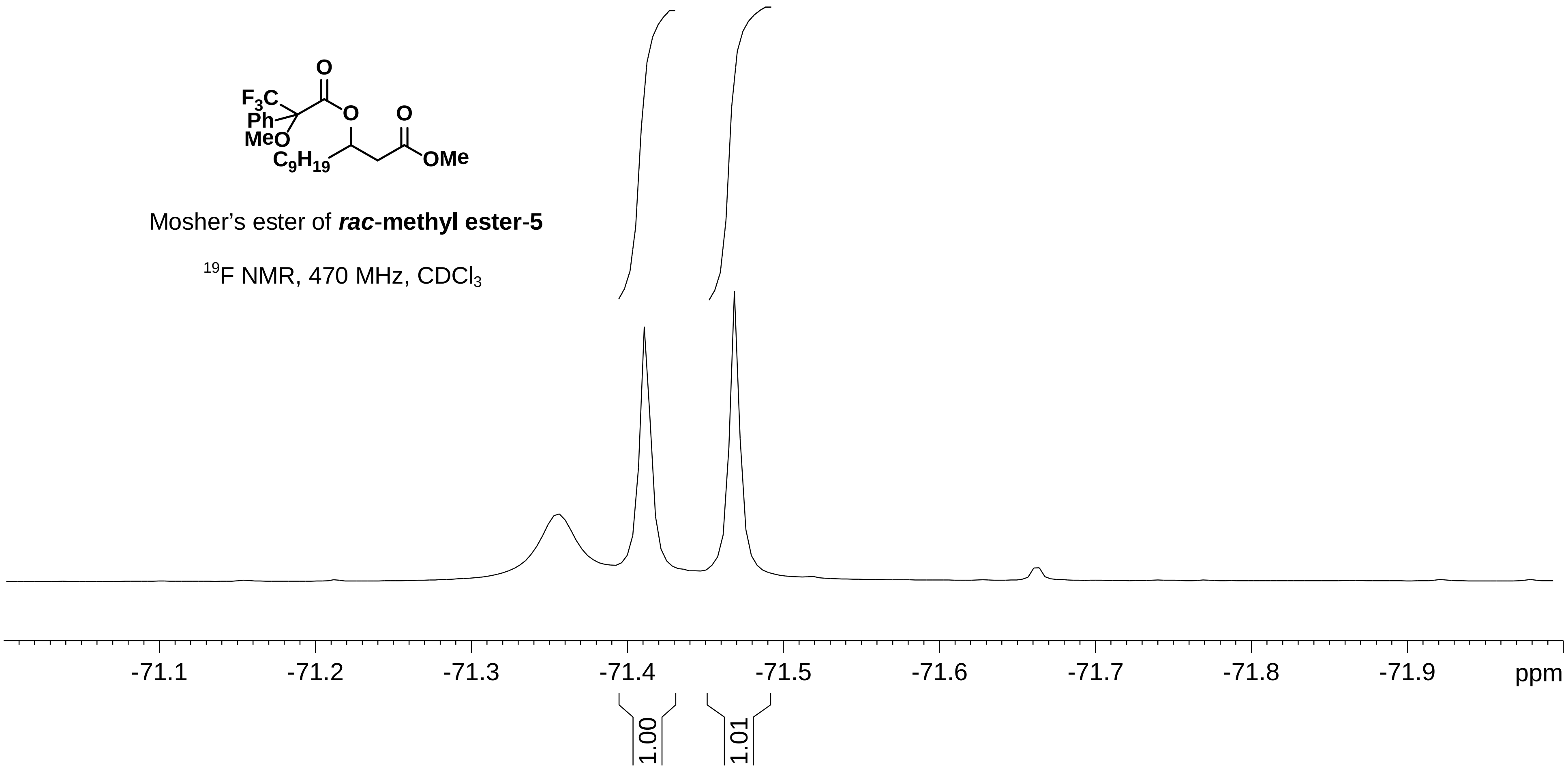

Figure S2. *Cont.*

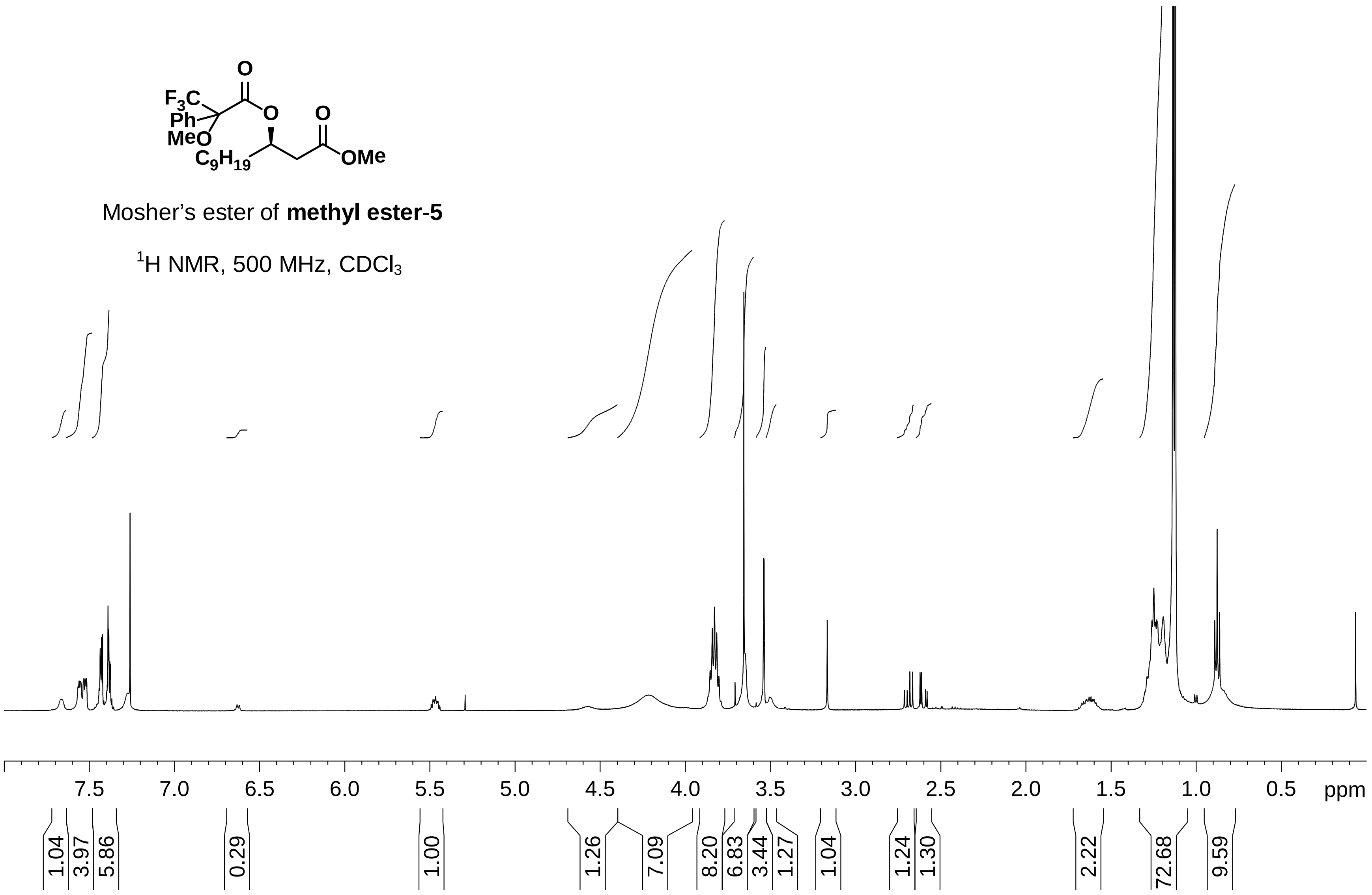

Figure S2. *Cont.*

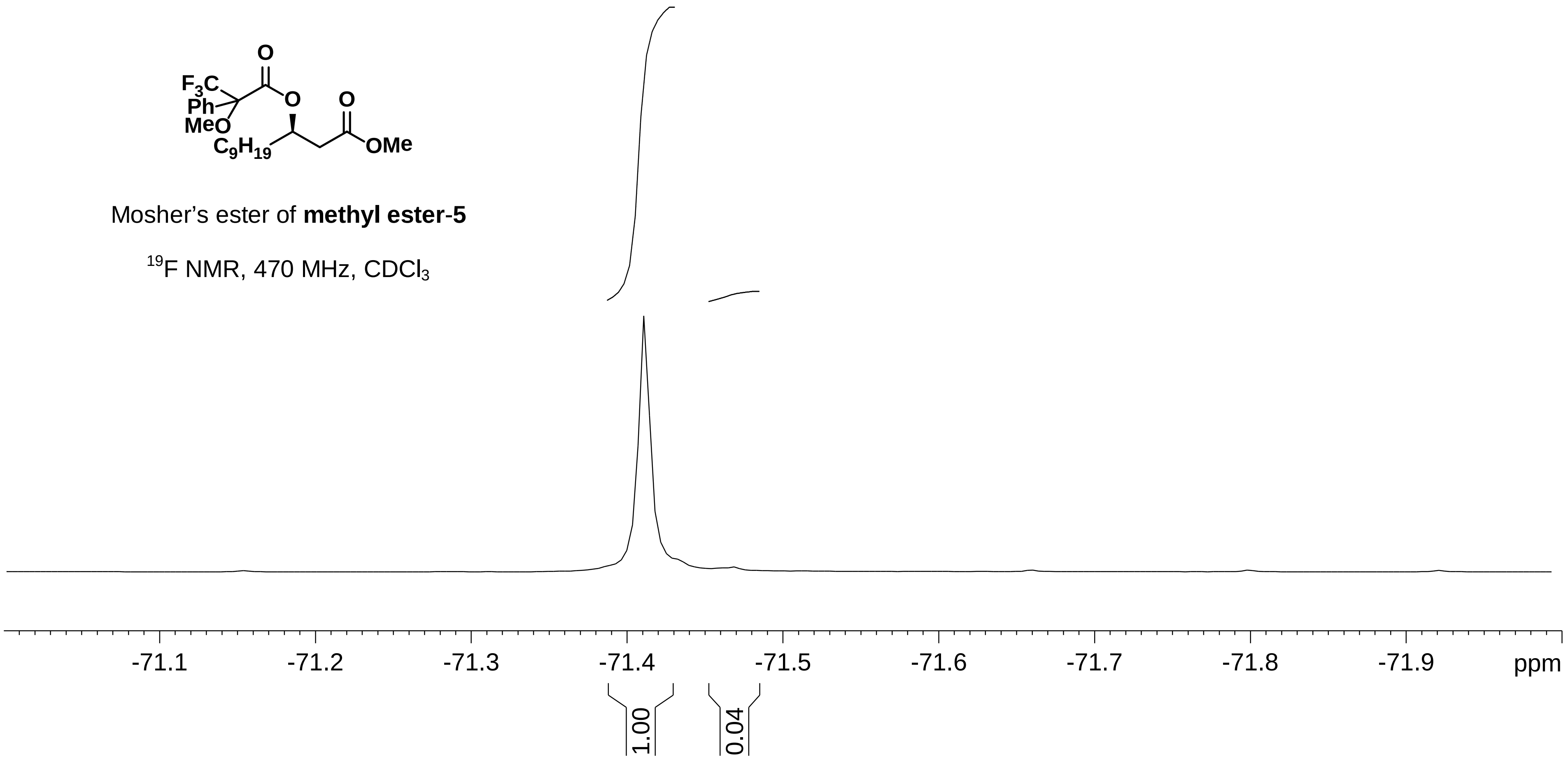

Figure S3. Comparison of ^{13}C NMR data between synthesized and natural fellutamide B (**2**). Synthesised fellutamide B; Synthetic: 125 MHz, d_6-DMSO; Natural: 101.6 MHz, d_6-DMSO.

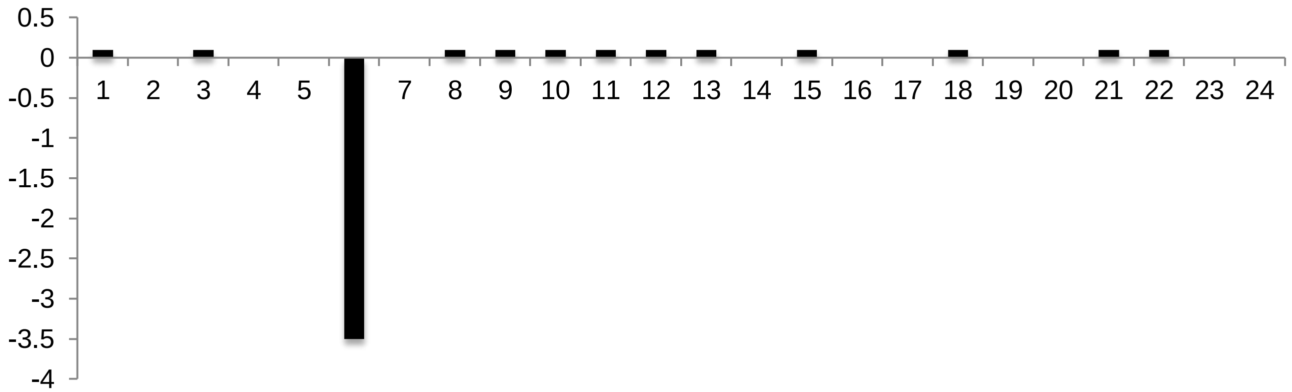

Figure S4. Raw data for *in vitro* inhibition assays against *Mycobacterium tuberculosis*.

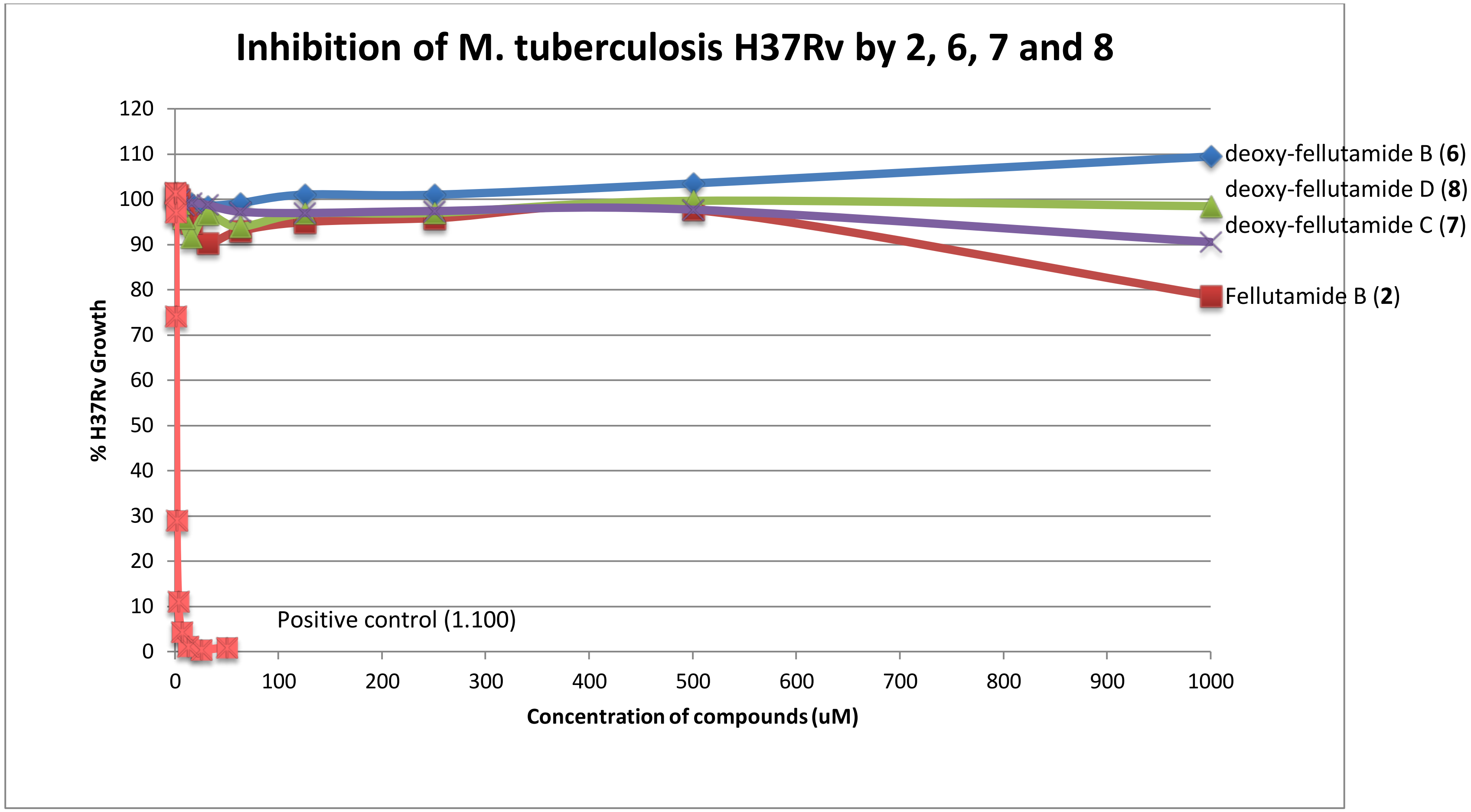

Supplement: Supplementary File 1 — Supplementary Material (PDF, 1369 KB) [file marinedrugs-11-02382-s001.pdf]
